# Equivalent Latitude Computation Using Regions of Interest (ROI)

**DOI:** 10.1371/journal.pone.0072970

**Published:** 2013-09-25

**Authors:** Juan A. Añel, Douglas R. Allen, Guadalupe Sáenz, Luis Gimeno, Laura de la Torre

**Affiliations:** 1 Smith School of Enterprise and the Environment, University of Oxford, Oxford, United Kingdom; 2 EPhysLab, Facultade de Ciencias, Universidade de Vigo, Ourense, Spain; 3 Naval Research Laboratory, Washington, District of Columbia, United States of America; 4 Departamento de Física, Facultad de Ciencias, Universidad de Extremadura, Badajoz, Spain; University of Florida, United States of America

## Abstract

This paper introduces a novel algorithm to compute equivalent latitude by applying regions of interest (ROI). The technique is illustrated using code written in Interactive Data Language (IDL). The ROI method is compared with the “piecewise-constant” method, the approach commonly used in atmospheric sciences, using global fields of atmospheric potential vorticity. The ROI method is considerably more accurate and computationally faster than the piecewise-constant method, and it also works well with irregular grids. Both the ROI and piecewise-constant IDL codes for equivalent latitude are included as a useful reference for the research community.

## Introduction

In atmospheric sciences Equivalent Latitude (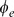) is a Lagrangian coordinate defined as the geographical latitude enclosing the same area as the isoline of a given atmospheric field on a 2D (longitude by latitude) surface [1], [2]. It is computed as:
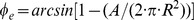


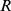
: radius of the sphere (e.g. Earth's radius).

Therefore computing 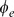 is simply the problem of computing the area 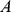 enclosed by the isoline of the studied property, which is essentially the determination of a function from a subset of data points.

One common technique to compute 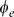 works by assuming the value of the atmospheric field to be constant within each grid cell (hereafter the ‘piecewise-constant’ method). To determine 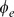 for a given threshold value of the field, you simply sum the areas for all grid cells with field values less than the threshold value.
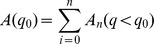


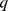
: value of the field. 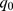: value to be evaluated. 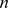: grid cell.

**Figure 1:**
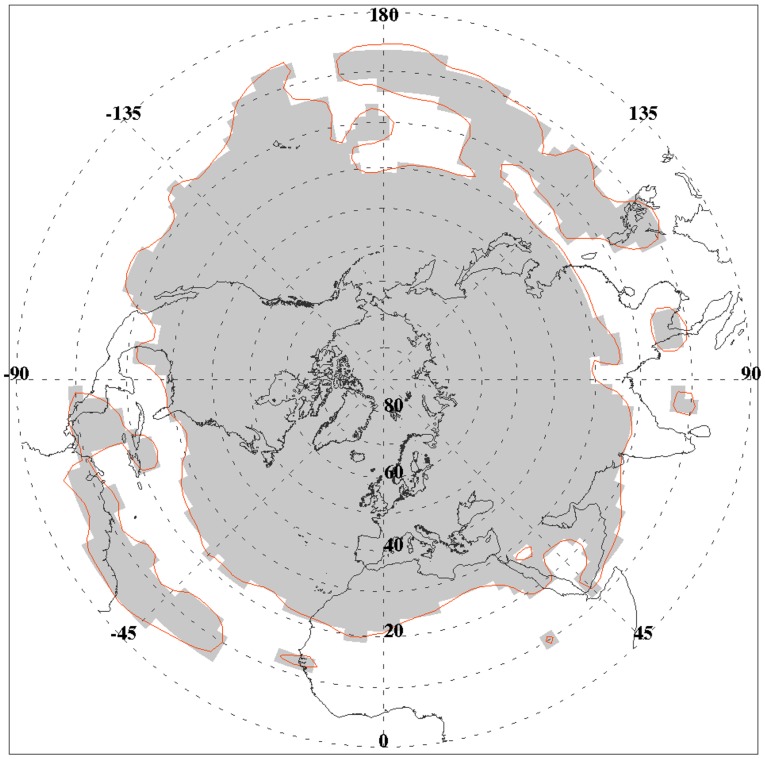
Comparison between the area used for the computation of 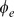 by the piecewise-constant method (gray surface) and the ROI technique (surface inside red contours) for a grid size of 2.5°×2.5°. The case corresponds to 1 January 1990 at OO UTC from NCEP1 for 2 PVU on an isentropic surface of 380 K.

The piecewise-constant approach is only first-order accurate, not allowing the atmospheric field to vary across the cell. Increased accuracy could be attained by higher-order approximations for the field variation (e.g., linear, parabolic, cubic interpolation). In this paper, we develop a higher-order solution to the problem of calculating 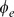 by using contour mapping techniques. This approach, known also as regions of interest (ROI), is a well known concept in medical imaging and is also used for geographical information systems. The general approach involves defining a region from a field (up to 4D), which is selected for a posteriori analysis. That is, given a field or sample, a subsample is selected which meets certain properties and that is the region in which we are interested for further analysis. Here we limit the field variation to the surface of a sphere (2D). The use of ROI based on interpolated contours is more accurate than the piecewise-constant method, since it is a better approximation to the real function (exact area). This is illustrated in figure 1, which maps the areas enclosed by a threshold value of potential vorticity (PV) using both the piecewise-constant and ROI methods. This is an advantage when considering, for example, small isolated and closed contours, since the size of the grid becomes more important in order to take them into account or not. For example, the ROI method is more accurate to assess the real area of a closed contour when it is slightly smaller or bigger than a cell or subset of cells of the grid.

In this paper we explore the ROI technique for the computation of 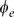 and compare the performance and accuracy with that obtained using the piecewise-constant method. For the purposes of this paper the ROI will be a 2D field of atmospheric PV [3], usually measured in PV units (PVU), where 
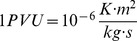
. The reason for this choice is that computation of 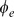 from PV is commonly used in atmospheric sciences, applied for example to the study of the stratospheric polar vortex [4], [5], and the goal for which we originally developed this approach. The differences between both techniques and, explained in a basic way, the steps that we follow are:

- piecewise-constant technique (traditional):
  1. PV is assumed to be constant within each grid cell;
  2. compute the area of each cell which value is less than the PV threshold that we are interested in;
  3. sum the area of the cells from step 2
- ROI technique:
  1. PV is ‘not’ constant within each grid cell;
  2. make the contour mapping function ‘draw’ (interpolate) the isolines of PV;
  3. compute the area enclosed by the isoline corresponding to the PV threshold value that we are interested in;

We developed the ROI code using the Interactive Data Language (IDL), a programming language extensively used for research in atmospheric sciences [6], and compare with piecewise-constant code also written in IDL. In this way we put the focus on the code and the technique, and do not take into account any dependence on programming language, corresponding libraries, or dependencies on hardware.

The following section describes the data used followed by the design and implementation of the ROI routine, addressing the ways in which we have solved the shortcomings of the computer language and subroutines affecting our implementation. This is followed by a results section that details the accuracy and performance of the ROI method relative to the piecewise-constant method. To conclude, we briefly discuss several ways of improving the solution here proposed, including using another programming languages and language interpreters.

## Materials and Methods

### Previous code and data

**Figure 2:**
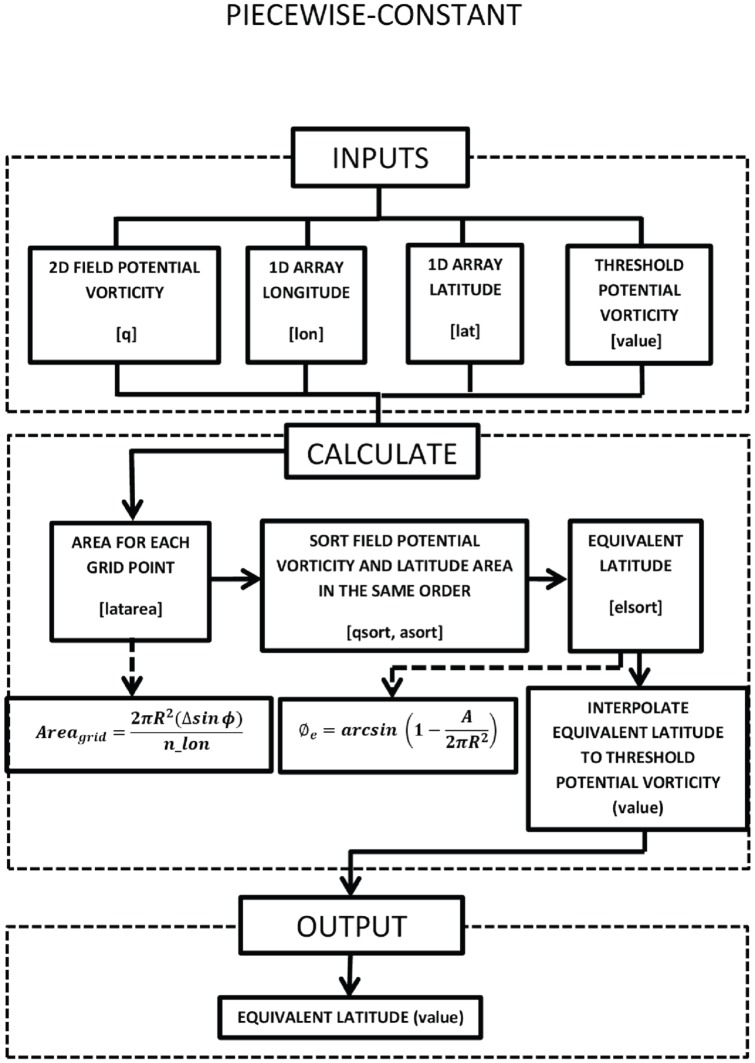
Flow diagram corresponding to the piecewise-constant code.

Routines to compute 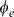 are available through internet, e.g. the ones using the NCAR Command Language (http://www.ncl.ucar.edu/Applications/equiv_lat.shtml) or pascal (http://www.bodekerscientific.com/how-do-i/calculate-equivalent-latitude). To check our new methodology against the more traditional one, we use the code (not previously published) supplied as (file S1) that applies the piecewise-constant method. This code takes as input a global gridded field with supplied latitude and longitude grids along with a desired threshold value of the field at which 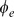 is calculated. The grid cells are sorted by the value of the field in each cell, and 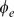 is calculated for every grid point on the globe. 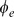 is then interpolated to the desired threshold value and is output to the user. A flow diagram is provided in figure 2.

We have tested our new ROI routine using fields of PV computed for five sets of reanalyzed data (NCEP/NCAR (hereafter NCEP1) [7], NCEP/DOE (hereafter NCEP2) [8], JRA-25 [9], ERA-Interim [10]), MERRA [11] and one chemistry-climate model (WACCM3 [12]). Additional information about the computation of the fields of PV, the datasets and results obtained with this routine can be obtained from Añel et al. [13].

### Design and Implementation

The method here proposed is based on a simple idea: given the field that we want to study (PV), we just need to plot it using a global contour map and then to make use of the IDL tools to detect what parts of the field meet a criterion (in our case that the value is below a given PVU threshold). Then the area that meets the criterion is automatically computed by the ROI detection tools avilable in IDL and we just need to apply the corresponding equation to get 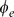.

**Figure 3:**
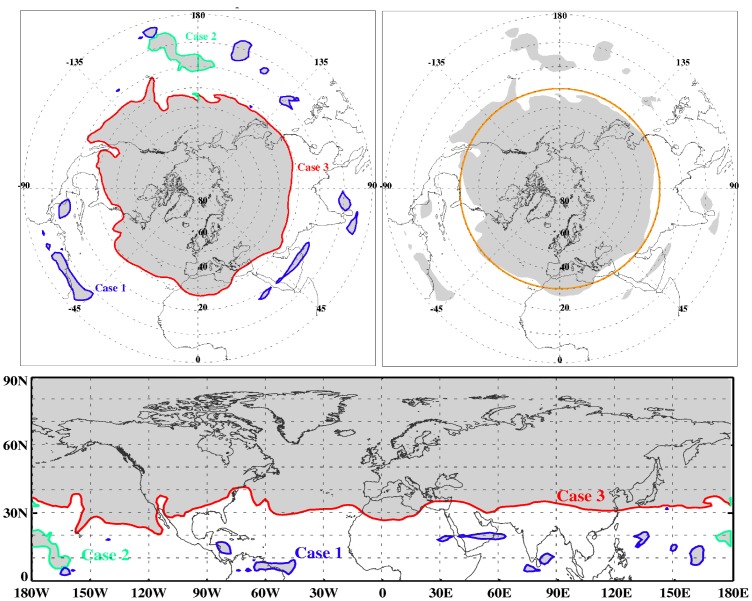
Upper left: global isosurface of 4 PVU on 1 January 1990 from ERA-Interim reanalysis data. Upper right: 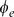 corresponding to the area covered by the 4 PVU isosurface (in this case 30.41°). Lower panel: cylindrical projection corresponding to the upper left panel.

The first problem to be solved for the implementation of the computation of the area using ROI is to determine the configuration of a given isoline of a global field projected on a sphere (the planet). Depicting the field with a cylindrical projection, an isosurface of PV can show three different configurations, directly related to the shape of the isolines over this projection (see figure 3):

- case 1: contours closed for any region of the planet, the easiest problem as it requires just applying the computation of the area using the available routines for ROI;
- case 2: contours going through the meridian 180 and not enclosing the pole are not closed in a cylindrical projection, so they need to be split in two different contours (one in each hemisphere) and the area has to be computed as the sum of the two different areas;
- case 3: the contours around the poles are not closed in a cylindrical projection. They go from the the lowest to the highest value in longitude. They are closed using a large number of points (resolution of 0.1°) at the latitude near the pole (North or South) and then the enclosing area is calculated using routines for ROI.

Another problem addressed during the design of this code was that the computation of ROI in IDL gives erroneous negative values if one line of the isolines of PV drawn by the function ‘contour’ crosses itself. This happens mainly for latitudes near to the poles in rectangular grids where cell size and isolines get narrow and the IDL ROI implementation fails for unknown reasons. We solve this problem by increasing the latitudinal resolution in these regions, studying the ROI with a resolution of 0.1° to avoid the problem of crossing isolines.

It was also necessary to create new matrix of data in order to close the contours not closed in the cylindrical projection, just because for example they cut the meridian zero as we mentioned before. An additional problem was discovered for the computation of cosines for 90° and −90°. IDL presents a bug and the results are negative values instead of zero. To avoid this problem we close the contours in a latitude of 89.99° instead of 90°.

The IDLanROI class is used to determine the regions enclosing specified threshold values of PV, and it is combined with the application of the ‘path_info’ keyword in the Contour procedure. Also we had to take into account the difference between computation of the geometric area (the real value of the mathematical calculation) and the masked region (value from the pixels in the displayed area) when using ROI in IDL. Therefore we applied the ComputeGeometry function to obtain the geometric area. ComputeGeometry relies on the Contour procedure to get the function.

Finally, the total area to be passed to the routine to compute the equivalent latitude is considered as the addition of the values for the different partial areas obtained. We use −9999.99 for the total area when (according to ‘path_info’) there is not a corresponding contour for the threshold PV value that we try to evaluate.

**Figure 4:**
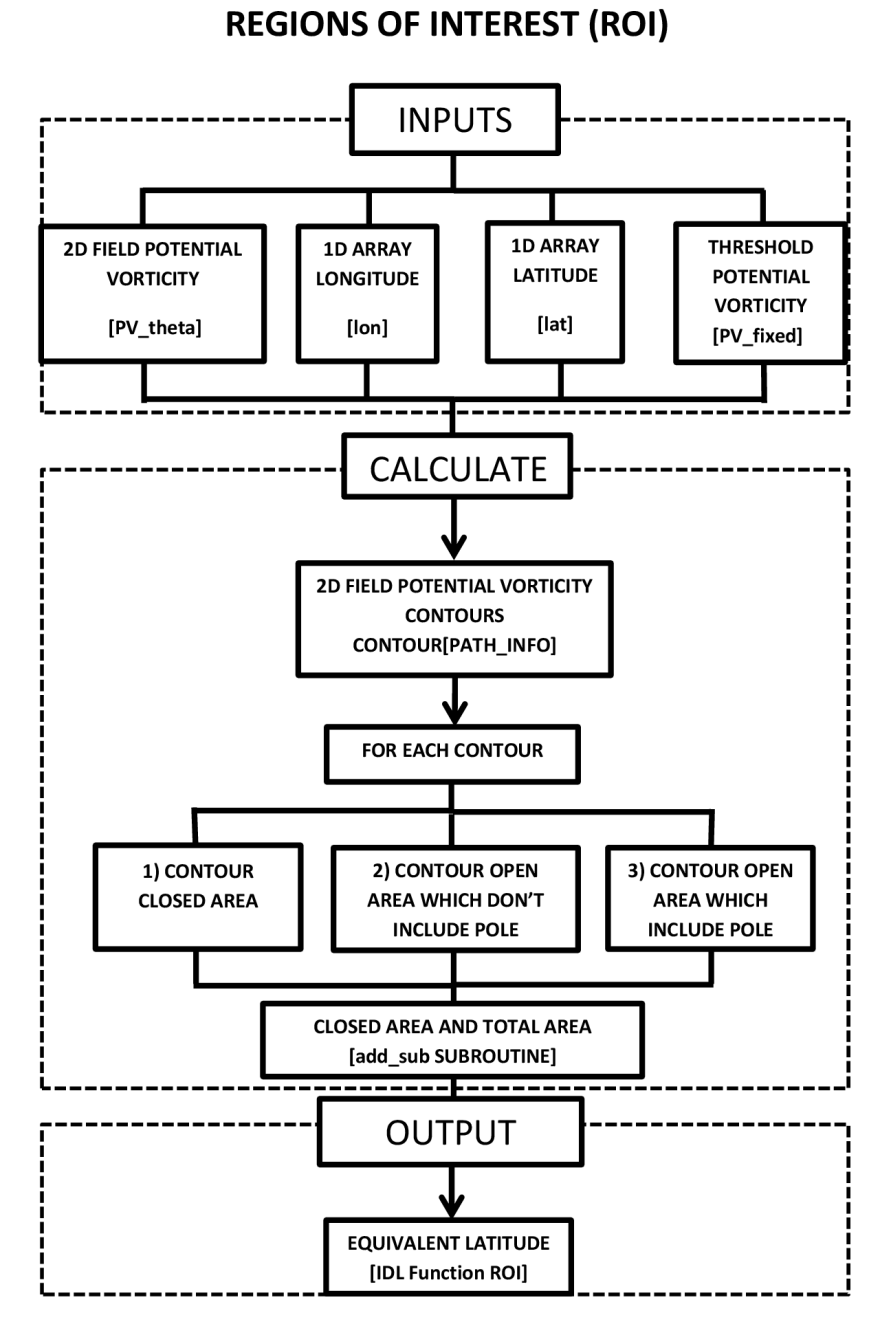
Flow diagram corresponding to the ROI code.

The process is explained in a flow diagram in the figure 4 and the ROI routine is included in file S2.

## Results

### Precision

**Figure 5:**
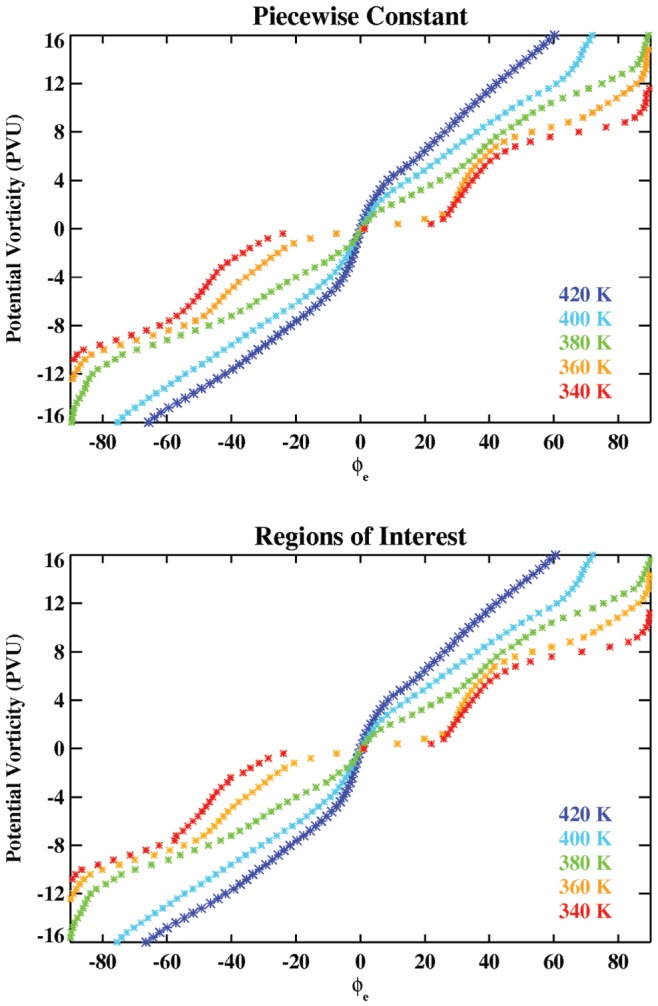
Comparison of PVU values for several isentropic surfaces and corresponding to different 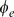. Values computed using the piecewise-constant and ROI methods on 1 January 1990 from ERA-Interim data.

First, we checked that the ROI methodology produces reasonable results for 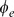 at all latitudes and for different isentropic surfaces. Figure 5 shows 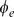 calculated from ERA-Interim PV data on 1 January 1990 using both the piecewise-constant and ROI methods from 340 to 420 K potential temperature. It is clear that the two methods produce very similar results, although there are some differences (e.g., near 40°S at 340 K).

**Figure 6:**
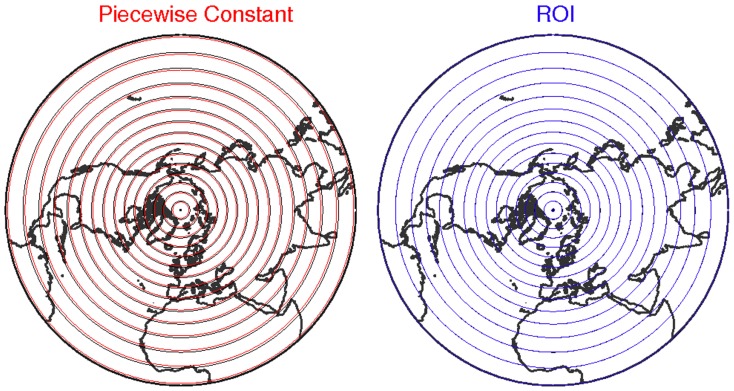
Red and blue circles: 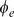 circles computed applying the piecewise-constant method and the ROI technique, respectively, from an ideal field of PV represented on a 2.5°×2.5° grid. Black circles: real values of 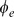 for the PV field studied (not seen for the case of the ROI technique as the blue ones are superimposed as they match perfectly the ideal field).

**Figure 7:**
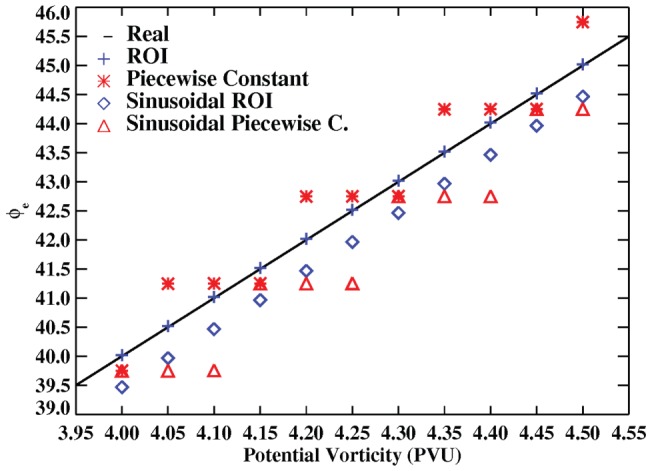
Accuracy of the ROI technique and the piecewise-constant method for different values of PV and latitude and for the two analytical functions.

Next, in order to check the accuracy of both methods we computated 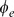 for two different cases in which the PV field is not based on real data, but rather on an analytic function (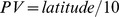 and 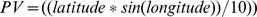, so that we know the exact relationship between 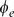 and PV. For the first case, the PV isolines form perfect circles around the pole and 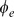 is simply equal to PV*10. In the second case we simply obtain a wavy field, but the relationship between 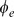 and PV is the same. The PV fields generated in this way are then used as input for both methods and the results are compared. Figure 6 shows the results using the piecewise-constant and ROI routines for the first method and figure 7 for both of them. Also the tables 1 and 2 quantify the variation of the error for different grid sizes. As it can be seen for a grid of 1.5°×1.5° the piecewise-constant method shows a bias respect to the corresponding 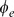 around a half degree for the first analytic function and 0.75 for the second one. This is greater than the bias for the ROI technique. Moreover the accuracy of the piecewise-constant method depends much more on the size of the grid (see both tables and figure 7).

**Table 1:** Precision dependence with technique and grid size in degrees for the PV field given by the first analytical function.

	ROI	piecewise-constant
1,5°×1,5°	0.030±0.025	0.415±0.237
3°×3°	0.027±0.015	0.659±0.384
4,5°×4,5°	0.029±0.020	1.145±0.660
6°×6°	0.029±0.022	1.497±0.892

Mean absolute values computed as the difference between the 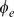 given by each technique and the real one. Average and standard deviation resulting of the computation for 50 different PV isolines (25 in the Northern Hemisphere and 25 in the Southern Hemisphere).

**Table 2:** Precision dependence with technique and grid size in degrees for the PV field given by the second analytical function.

	ROI	piecewise-constant
1,5°×1,5°	0.540±0.010	0.746±0.415
3°×3°	0.877±0.010	0.920±0.676
4,5°×4,5°	0.801±0.010	1.255±0.836
6°×6°	0.801±0.010	1.563±1.022

Mean absolute values computed as the difference between the 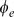 given by each technique and the real one. Average and standard deviation computed following the same method that for table 1.

### Performance

As we can see in tables 3 and 4 the ROI technique computed in this way is faster than the piecewise-constant method for a grid of 1.5°×1.5°, and moreover the finer the grid the greater the advantage in computational time for the ROI technique. This is something to have into account as in the future we probably will be moving to smaller grid sizes. This result is probably because the piecewise-constant method relies for the evaluation of the values of the function in each point on several FOR loops, which makes especially slow any computation in IDL. However the ROI technique simply computes the function once.

**Table 3:** Computation time dependence with technique and grid size in 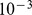 seconds for the PV field given by the first analytical function.

	ROI	piecewise-constant
1,5°×1,5°	4.09±0.16	36.97±0.25
3°×3°	1.33±0.18	9.16±0.03
4,5°×4,5°	0.83±0.18	4.18±0.02
6°×6°	0.66±0.18	2.39±0.01

Average and standard deviation computed following the same method that for table 1.

**Table 4:** Computation time dependence with technique and grid size in 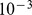 seconds for the PV field given by the second analytical function.

	ROI	piecewise-constant
1,5°×1,5°	4.13±0.17	43.13±0.79
3°×3°	1.35±0.17	10.57±0.10
4,5°×4,5°	0.85±0.17	4.82±0.04
6°×6°	0.68±0.17	3.16±0.03

Average and standard deviation computed following the same method that for table 1.

The test was performed on a computer with the following hardware: Intel Quad 2 Core with cpu frequency of 2.66 GHz and 3 MB of cache memory, 3 GB of RAM memory. The operative system was Ubuntu GNU/Linux i686 with Linux kernel 2.6.32–37-generic. The IDL interpreter was provided by EXELIS and the version was 8.0.

## Availability and Future Directions

The ROI code described in this paper is publicly available from the URL https://github.com/eqlat/roi. As expressed in the code of the routine the code is under the GNU General Public License (GPL) (http://www.gnu.org/licenses). In this way we comply with the criteria on public availability of code for research purposes [14]. To work with it, it is just necessary to get the code and to have and IDL interpreter.

We suggest as potential future directions for development the possibility of writing an equivalent ROI code in Python or Fortran, two programming languages very used in the geociences community, but it should been had into account that very limited support for ROI computation seems to exist in both languages. To the authors knowledge just the NiPy library (http://nipy.sourceforge.net) for Python, currently under development, and some punctual implementations in Fortran. Also, currently is not possible to use the code based on ROI with GNUdatalanguage (http://gnudatalanguage.sourceforge.net), a free IDL compiler, as computation of ROI is not implemented in it.

## Supporting Information

File S1 **Piecewise-constant code.**
(PDF)

File S2 **ROI code.**
(PDF)
