# Supplementary material for: Equivalent Latitude Computation Using Regions of Interest (ROI)
Source: PLoS One. 2013 Sep 25;8(9):e72970. doi: 10.1371/journal.pone.0072970 (PMC3783454; doi:10.1371/journal.pone.0072970)
Supplement: File S1 — Piecewise-constant code. (PDF) [file pone.0072970.s001.pdf]

```
;*********************************************************************************************

;************************************************************
; This program calculates equivalent latitude for a given  |
; 2-D field. The user inputs the 2-D field along with the  |
; longitude and latitude, which must be on evenly-spaced   |
; grids. The latitude grid may either start/and at the     |
; poles or be staggered 1/2 grid point from the poles.     |
; This is checked by the min value of lat. If min(lat) is  |
; -90. then the grid begins and ends at the pole. The      |
; longitude grid must be evenly spaced (with spacing dlon) |
; and can either go from 0 to 360-dlon or from dlon/2 to   |
; 360-dlon/2.                                              |
;                                                          |
; Last modified August 2, 2001                             |
;                                                          |
; Please report any bugs to Douglas Allen                  |
;************************************************************

function calcelat2d, q, lon, lat, value

nlon=n_elements(lon)         ; regularly-gridded latitude (degrees)
nlat=n_elements(lat)         ; regularly-gridded longitude (degrees)
dlat=lat(1)-lat(0)           ; latitude grid spacing
elat2d=FltArr(nlon,nlat)     ; equivalent latitude on 2D grid
a=6.37e3                     ; radius of earth in km
hemarea=2.*!pi*a^2           ; surface area of hemisphere in km^2
latarea=FltArr(nlat)         ; area of grid point at each latitude

;------ Calculate area for each grid point ------

if(min(lat) eq -90.) then begin
  ;------ End grid points on the poles ------

  phi1=!pi/180.*(-90.)
  phi2=!pi/180.*(-90.+dlat/2.)
  latarea(0) = hemarea*abs(sin(phi1)-sin(phi2))/float(nlon)

  for i=1,nlat-2 do begin
    phi1=!pi/180.*(lat(i)-dlat/2.)
    phi2=!pi/180.*(lat(i)+dlat/2.)
    latarea(i) = hemarea*abs(sin(phi1)-sin(phi2))/float(nlon)
  endfor

  phi1=!pi/180.*(90.-dlat/2.)
  phi2=!pi/180.*(90.)

  latarea(nlat-1) = hemarea*abs(sin(phi1)-sin(phi2))/float(nlon)

endif else begin

  ;------ End grid points 1/2 grid off the poles ------

  for i=0,nlat-1 do begin
    phi1=!pi/180.*(lat(i)-dlat/2.)
    phi2=!pi/180.*(lat(i)+dlat/2.)
    latarea(i) = hemarea*abs(sin(phi1)-sin(phi2))/float(nlon)
  endfor
endelse

totalarea=total(latarea)*nlon

;------ order the data points by increasing value ------

npoints=long(nlon)*long(nlat)

q1d=FltArr(npoints)                 ; Data placed in 1-D array
a1d=FltArr(npoints)                 ; Area placed in 1-D array
el1d=FltArr(npoints)                ; Elat placed in 1-D array

index=long(0)
for ilon=0,nlon-1 do begin
  for ilat=0,nlat-1 do begin
```

```
    q1d(index)=q(ilon,ilat)
    a1d(index)=latarea(ilat)

    index=index+1
  endfor
endfor
qsort=q1d(sort(q1d))               ; Sorted data
asort=a1d(sort(q1d))               ; Sorted area

tasort=FltArr(npoints)             ; Total area for sorted mixing ratio
elsort=FltArr(npoints)             ; Equivalent latitude for sorted mr

ta=0.                              ; Total area

for index=long(0),npoints-1 do begin
  ta=ta+asort(index)
  if(ta gt 2.*hemarea) then ta=2.*hemarea ; keep area le 2.*hemarea
  tasort(index)=ta
  elsort(index)=asin(ta/hemarea-1)/!dtor
endfor

latitequival=interpol(elsort(*),qsort(*),value)

return, latitequival

;***********************************************************************************************

end
```
