# Supplementary material for: Equivalent Latitude Computation Using Regions of Interest (ROI)
Source: PLoS One. 2013 Sep 25;8(9):e72970. doi: 10.1371/journal.pone.0072970 (PMC3783454; doi:10.1371/journal.pone.0072970)
Supplement: File S2 — ROI code. (PDF) [file pone.0072970.s002.pdf]

```
;-----------------------------------------------------------------------------------------------------------
;Copyright (C) 2013  Juan Antonio Añel, Guadalupe Sáenz, Laura de la Torre and Doug Allen.
;
;    This program is free software: you can redistribute it and/or modify
;    it under the terms of the GNU General Public License as published by
;    the Free Software Foundation, either version 3 of the License, or
;    (at your option) any later version.
;
;    This program is distributed in the hope that it will be useful,
;    but WITHOUT ANY WARRANTY; without even the implied warranty of
;    MERCHANTABILITY or FITNESS FOR A PARTICULAR PURPOSE.  See the
;    GNU General Public License for more details.
;
;    You should have received a copy of the GNU General Public License
;    along with this program.  If not, see
;    <http://www.gnu.org/licenses/>.
;-----------------------------------------------------------------------------------------------------------
;
;NAME: eqlat_roi
;
;PURPOSE:
;
;     This program calculates equivalent latitude using the concept of regions of interest (ROI)
;     for 2D field of Potential Vorticity (PV).
;
;CATEGORY:
;
;     Physic
;
;INPUTS:
;
;     PV_theta: Potential Vorticity field for a fixed isentropic surface (units PVU).
;     lon: longitude grid of Potential Vorticity field (-180 to 180).
;     lat: latitude grid of Potential Vorticity field (90 to -90).
;     PV_fixed: umbral Potential Vorticity for calculate equivalent latitude (units PVU).
;
;OUTPUTS:
;
;     latequival: equivalent latitude in degree for a fixed Potential Vorticity (PV_fixed).
;
;EXAMPLE:
;
; IDL> lon=findgen(145)*2.5-180.
; IDL> lat=findgen(73)*2.5-90.
; IDL> PV_fixed=4
; IDL> eqlat_roi,PV_theta,lon,lat,PV_fixed,equivalat
; IDL> print, equivalat
; IDL> -26.505798
;
;AUTHORS AND MODIFICATIONS
;     Last modified July 26, 2013 Juan Antonio Anel, Guadalupe Saenz Laura de la Torre and Doug Allen.

;Please report any bugs to Juan Anel (juan.anel@smithschool.ox.ac.uk) and Guadalupe Saenz
(lusaga81@gmail.com).
;***********************************************************************************************************

pro eqlat_roi,PV_theta,lon,lat,PV_fixed,equivalat,time_a
timer,/start
;----------
constant-----------------------------------------------------------------------------------------------
RT=6.37e3; Earth Radius (in meters)

;------------Closed PV_theta
field-----------------------------------------------------------------------------
if (lon(n_elements(lon)-1) lt 180) then begin

   PV_theta11=fltarr(n_elements(lon)+1,n_elements(lat))
   PV_theta11(0:n_elements(lon)-1,*)=PV_theta(*,*)
   PV_theta11(n_elements(lon),*)=PV_theta(0,*)
   lon1=fltarr(n_elements(lon)+1)
   lon1(0:n_elements(lon1)-2)=lon
   lon1(n_elements(lon1)-1)=lon(0)+360
   lat11=lat
endif else begin
```

```
   PV_theta11=PV_theta
   lon1=lon
   lat11=lat
endelse
;-----------Latitude (90,-90)---------------------------------------------------------------------
PV_theta1=fltarr(n_elements(lon1),n_elements(lat11))
if lat11(0) lt lat11(1) then begin
   lat1=reverse(lat11)
   for i=0,n_elements(lon1)-1 do begin
     PV_theta1(i,*)=reverse(reform(PV_theta11(i,*),n_elements(lat11)))
   endfor
endif else begin
   lat1=lat11
   PV_theta1=PV_theta11
endelse
;-----------------------Computation of
contours------------------------------------------------------------------
contour, PV_theta1,lon1,lat1,levels=PV_fixed,close=0,path_xy=xy,path_info=info,/path_data_coords,/
path_double
;-------------------Calculate areas of
interes-------------------------------------------------------------------
totalarea=-9999.9
if n_elements(info) eq 0 then goto,no ; there aren't contours
totalarea=0.0
      for interesofarea=0,n_elements(info)-1 do begin ; there are contours
;---------------------------------------------------------------------------------------------------------
;---------An isosurface of PV can show three different cases of computation of areas-------------------
               ;------------------------------------------------------------------------------------------
               ;Case 1// Closed areas.
               ;Case 2// Open areas which don't include pole.
               ;Case 3// Open areas which include pole.
;---------------------------------------------------------------------------------------------------------
 a=info(interesofarea).offset & b=info(interesofarea).offset+info(interesofarea).N-1

;Case 1// Closed areas
;---------------------------------------------------------------------------------------------------------
;In this case the contour is closed with one point (x_inicial=x_final and y_inicial=y_final)
;and the area enclosed is calculated with the function ROI.
;---------------------------------------------------------------------------------------------------------
   if (info(interesofarea).type eq 1) then begin
        x=fltarr(info(interesofarea).N+1)
        y=fltarr(info(interesofarea).N+1)
        x(0:info(interesofarea).N-1)=xy(0,a:b)
        y(0:info(interesofarea).N-1)=xy(1,a:b)
        x(info(interesofarea).N)=x(0)
        y(info(interesofarea).N)=y(0)
   add_sub,x,y,x,y,0,lon1,lat1,PV_theta1,PV_fixed,partialarea
   totalarea=totalarea+partialarea
   endif
   x=0
   y=0
; Open contour
   if (info(interesofarea).type eq 0) then begin

        x=fltarr(info(interesofarea).N)
        y=fltarr(info(interesofarea).N)
        x(0:info(interesofarea).N-1)=xy(0,a:b)
        y(0:info(interesofarea).N-1)=xy(1,a:b)
;Case 2// Open contour which don't include pole
;---------------------------------------------------------------------------------------------------------
;The contour is closed with one point (x_inicial=x_final and y_inicial=y_final)
;and the area enclosed is calculated with the function ROI.
;---------------------------------------------------------------------------------------------------------
      if (x(0) eq x(n_elements(x)-1)) then begin
        xcl=fltarr(n_elements(x)+1) & ycl=fltarr(n_elements(y)+1)
        xcl(0:n_elements(x)-1)=x    & ycl(0:n_elements(y)-1)=y
        xcl(n_elements(x))=x(0)
        ycl(n_elements(y))=y(0)

        add_sub,xcl,ycl,x,y,0,lon1,lat1,PV_theta1,PV_fixed,partialarea
        totalarea=totalarea+partialarea
   endif else begin

;Case 3// Open contour which include pole
```

```
;-----------------------------------------------------------------------------------------------------------
;The contour is closed using a lot of points with high resolution of 0.1 degrees
;of latitude to the pole (North or South).
;Finally the enclosing area is calculated using function ROI.
;-----------------------------------------------------------------------------------------------------------
        if PV_fixed ge 0 then begin
           beginningpoints=abs(1.0*floor(10.0*(90-(y(0)))))
           finalpoints=abs(1.0*floor(10.0*(90-y(n_elements(y)-1))))
        endif
        if PV_fixed lt 0 then begin
           beginningpoints=abs(1.0*floor(10.0*(-90-(y(0)))))
           finalpoints=abs(1.0*floor(10.0*(-90-y(n_elements(y)-1))))
        endif
        if beginningpoints eq 0 or beginningpoints eq 1 then beginningpoints=2
        if finalpoints eq 0 or finalpoints eq 1 then finalpoints=2
        totalpoints=beginningpoints+finalpoints
        xclosed=fltarr(n_elements(x)+totalpoints+2) & yclosed=fltarr(n_elements(y)+totalpoints+2)
        xclosed(0:beginningpoints-1)=x(0)
              if PV_fixed ge 0 then begin
                yclosed(0)=90.
                yclosed(1:beginningpoints-1)=90.-indgen(beginningpoints-1)*0.1
              endif
              if PV_fixed lt 0 then begin
                yclosed(0)=-90.
                yclosed(1:beginningpoints-1)=-90.+indgen(beginningpoints-1)*0.1
              endif
        xclosed(beginningpoints:beginningpoints+n_elements(x)-1)=x
        yclosed(beginningpoints:beginningpoints+n_elements(y)-1)=y
        xclosed(beginningpoints+n_elements(x):n_elements(xclosed)-2)=x(n_elements(x)-1)
                if (PV_fixed ge 0) then yclosed(beginningpoints+n_elements(y):n_elements(yclosed)-2)$
                =reverse(89.99-indgen(finalpoints+1)*0.1)
                if (PV_fixed lt 0) then yclosed(beginningpoints+n_elements(y):n_elements(yclosed)-2)$
                =y(n_elements(y)-1)-indgen(finalpoints+1)*0.1
        xclosed(n_elements(xclosed)-1)=xclosed(0)
                if PV_fixed ge 0 then yclosed(n_elements(yclosed)-1)=90.
                if PV_fixed lt 0 then yclosed(n_elements(yclosed)-1)=-90.
        add_sub,xclosed,yclosed,x,y,beginningpoints,lon1,lat1,PV_theta1,PV_fixed,partialarea
;----- sum of total area-----------------------------------
        totalarea=totalarea+partialarea
;--------------------------------------------------------------
      endelse
   endif
endfor
;----------------------------Calculate equivalent latitude-----------------------------------------
   latitudecalculate=equival_latit(totalarea)
        if PV_fixed ge 0 then begin
           equivalat=latitudecalculate*!radeg
        endif
        if PV_fixed lt 0 then begin
           equivalat=-(latitudecalculate*!radeg)
        endif
no:
if totalarea eq -9999.9 then begin
   equivalat=!values.f_nan
endif
timer,/stop,/print,time
time_a=time
return
end

;-------------------------------------------------------------------------------------------------------
;This function calculates what areas are added and/or subtracted.
;-------------------------------------------------------------------------------------------------------
pro add_sub,x1,y1,x2,y2,mas,lon_1,lat_1,PV_theta_11,PV_fix,partialarea1
    RT=6.37e3; Earth Radius (in meters)

       x_l=x1(mas+floor(n_elements(x2)/2.))
       y_l=y1(mas+floor(n_elements(x2)/2.))
       object1=Obj_New('IDLanROI',x1,y1)
       ind_lat=where(y_l ge lat_1,c_lat)
       ind_lon=where(x_l le lon_1,c_lon)

if(ind_lat(0) ne 0 and ind_lat(0) ne n_elements(lat_1)-1) then begin
  i_lat=[ind_lat(0)+1,ind_lat(0)-1]
```

```
endif else begin
  if (ind_lat(0) eq 0) then i_lat=ind_lat(0)+1
  if (ind_lat(0) eq n_elements(lat_1)-1) then i_lat=ind_lat(0)-1
endelse
if(ind_lon(0) ne 0 and ind_lon(0) ne n_elements(lon_1)-1) then begin
  i_lon=[ind_lon(0)+1,ind_lon(0)-1]
endif else begin
  if (ind_lon(0) eq 0) then i_lon=ind_lon(0)+1
  if (ind_lon(0) eq n_elements(lon_1)-1) then i_lon=ind_lon(0)-1
endelse


     pto=intarr(n_elements(i_lon),n_elements(i_lat))
     PV=fltarr(n_elements(i_lon),n_elements(i_lat))
     for ila=0,n_elements(i_lat)-1 do begin
       for ilo=0,n_elements(i_lon)-1 do begin
           pto0=lon_1(i_lon(ilo))
           pto1=lat_1(i_lat(ila))
           pto(ilo,ila)=object1->ContainsPoints(pto0,pto1)
           PV(ilo,ila)=PV_theta_11(i_lon(ilo),i_lat(ila))
       endfor
     endfor
       Obj_Destroy,object1
       exterior=where(pto eq 0,c_ext)
       interior=where(pto eq 1,c_int)
       if c_ext gt 0 and c_int gt 0 then begin
         PV_exterior=PV(exterior(0))
         PV_interior=PV(interior(0))

         xf=1d*x1*!dtor*RT*cos(y1*!dtor)
         yf=y1*!dtor*RT
         object = Obj_New('IDLanROI', xf, yf)
         res=object->ComputeGeometry(area=partialarea1)
         if partialarea1 lt 0 then partialarea1=-partialarea1
         Obj_Destroy, object
         if PV_fix ge 0 and PV_exterior gt PV_interior then partialarea1=-partialarea1
         if PV_fix lt 0 and PV_exterior lt PV_interior then partialarea1=-partialarea1
       endif
       if c_ext eq 0 and c_int gt 0 then begin
         PV_interior=PV(interior(0))
         xf=1d*x1*!dtor*RT*cos(y1*!dtor)
         yf=y1*!dtor*RT
         object = Obj_New('IDLanROI', xf, yf)
         res=object->ComputeGeometry(area=partialarea1)
         if partialarea1 lt 0 then partialarea1=-partialarea1
          Obj_Destroy, object
          if PV_fix ge 0 and PV_interior lt PV_fix then partialarea1=-partialarea1
          if PV_fix lt 0 and PV_interior gt PV_fix then partialarea1=-partialarea1
       endif
       if c_ext gt 0 and c_int eq 0 then begin
          PV_exterior=PV(exterior(0))
          xf=1d*x1*!dtor*RT*cos(y1*!dtor)
          yf=y1*!dtor*RT
          object = Obj_New('IDLanROI', xf, yf)
          res=object->ComputeGeometry(area=partialarea1)
          if partialarea1 lt 0 then partialarea1=-partialarea1
         Obj_Destroy, object
         if PV_fix ge 0 and PV_exterior gt PV_fix then partialarea1=-partialarea1
         if PV_fix lt 0 and PV_exterior lt PV_fix then partialarea1=-partialarea1
       endif

return
end

;---------------------------------------------------------------------
;This function calculates the equivalent latitude for the total area.
;---------------------------------------------------------------------
function equival_latit,area
  RT=6.371e3
  equivalatitude=asin(1-(area*1.0/(2*!pi*RT*RT)))
  if (abs(1-(area*1.0/(2*!pi*RT*RT))) gt 1) then begin
    print,'you can not obtain a value greater than 1'
  endif
return,equivalatitude
```

end
